# A Comparative Analysis of Standardised Threads for Use in Implants for Direct Skeletal Attachment of Limb Prosthesis: A Finite Element Analysis

**DOI:** 10.1155/2019/8027064

**Published:** 2019-02-07

**Authors:** Piotr Prochor, Eugeniusz Sajewicz

**Affiliations:** Department of Biocybernetics and Biomedical Engineering, Faculty of Mechanical Engineering, Bialystok University of Technology, Bialystok 15-351, Poland

## Abstract

The aim of the research was to determine the optimal thread's shape to be used in implants for direct skeletal attachment of limb prosthesis. In addition, by testing appropriate parameters' modification of the suitable thread, an attempt was made to maximise its effectiveness. The analyses included three thread types described in the ISO standards: shallow, symmetrical, and asymmetrical. The obtained results suggest that shallow thread ensures the lowest equivalent and directional stress peaks generated in the bone as well as favourable stress patterns and profiles during implant loading in relation to symmetrical and asymmetrical threads. Moreover, shallow thread ensured the generation of single equivalent and directional stress peaks, while symmetrical and asymmetrical threads provided additional stress peak for equivalent as well as for each of directional peaks. Subsequently, optimisation of the shallow thread's shape was conducted by changing two relevant thread's parameters (flank angle and rounding arc) which influence the generated stress distribution. The highest reduction of stress peaks was obtained while reducing the rounding arc by 0.2 mm. Therefore, it can be stated that the proposed modification of the HA thread can lead to obtaining a higher biomechanical effectiveness of implants for direct skeletal attachment of limb prosthesis.

## 1. Introduction

Traditionally, lower limb prostheses are connected to the stump with the use of an individually fitted socket. However, due to a number of disadvantages arising from their use, such as skin abrasions and poor prosthesis control resulting from the socket loosening, physicians and engineers have started to develop new limb-prosthesis connection solutions [1–6]. One of these solutions is implants for direct skeletal attachment (DSA) of limb prosthesis. These are specially designed constructions that are implanted into the marrow cavity of the bone, with a shaft penetrating soft tissues on which the external prosthesis is attached [1–7].

One way to connect the implant for direct skeletal attachment to the bone is to use a proper thread connection. An example of the implant that uses this method is the OPRA (Osseointegrated Prostheses for the Rehabilitation of Amputees) [5, 8]. It is currently the most common implant solution for direct limb prosthesis in bone fixation, which includes the use of the thread. Direct skeletal attachment of limb prosthesis is currently still developing connecting method being the reason for conducting proper analyses with the use of computer or experimental methods [9–16].

Applying thread as implant anchoring element may result in occurring stress peaks thatcan cause local bone resorption. As a result, it may lead to the loosening of an implant in a bone and the necessity of its removal. This problem has been reported in studies of threaded dental implants, which may suggest possible existence of the same problem in threaded implants for bone-anchored prostheses [17–21]. Stress peaks would explain the reason of the failure in some cases of the currently proposed construction solutions of implants for DSA [2]. The implant's thread should reduce the stress peaks as well as provide the highest implant-bone contact area in order to ensure appropriate secondary stabilisation [18, 22, 23]. However, so far, there are no analyses that optimise a proper thread which could be used in implants for bone-anchored prostheses. Few studies present results that could be used in the process of designing an implant thread for bone-anchored prostheses [17, 24]. During the development of implants, engineers can base on numerous research optimising the shape of the dental implant's thread [25, 26]. Nevertheless, due to the fact that the method of their attachment differs significantly from the attaching implant for direct skeletal attachment into the marrow cavity, there is no possibility of applying exactly the same thread design.

The aim of this study was to evaluate the efficiency of standardised threads, defined in appropriate standards, for use in implants for DSA of limb prosthesis. Three types of threads, commonly used in medical screws, were considered: HA: shallow thread; HC: symmetrical thread; HD: asymmetrical thread [27, 28]. Additionally, the authors attempted to modify the most appropriate standardised thread (which was determined during the analysis), in order to increase its efficiency in implant for DSA. The effectiveness of appropriate load transferring by the implants with the analysed threads was determined by comparing the stress patterns and profiles and stress peak values that were generated in the bone during different loading methods. The obtained results may allow increasing the functionality of currently used threaded implants for bone-anchored prostheses, as well as newly developed solutions.

## 2. Materials and Methods

### 2.1. Analysed Threads

**Figure 1:**
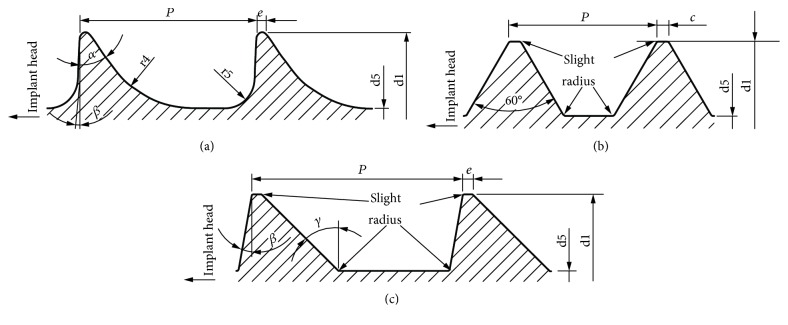
Thread types included in analyses. (a) HA: shallow thread [27]. (b) HC: symmetrical thread [28]. (c) HD: asymmetrical thread [28].

In the analyses, three types of standardised threads were considered (Figure 1), which are used mostly in medical screws. However, after suitable modifications, described threads find the use as threads of dental implants [25, 27, 28].

The highest shapes of threads that have been specified in the standards were chosen for analyses (individual dimensions were approximately similar to each other). The threads were HA 5, HC 4.2, and HD 4.5. HB thread, defined in ISO 5835:1991 standard, was omitted in analyses, as it is intended for use as an anchoring element in cancellous bone. Minor diameter of each thread was set as 19.5 mm. Slight radii considered in HC and HD threads were equal 0.025 mm. Undescribed threads' parameters are defined in the appropriate standards [27, 28].

### 2.2. Characteristics of Finite Element Models Created for Analyses

**Figure 2:**
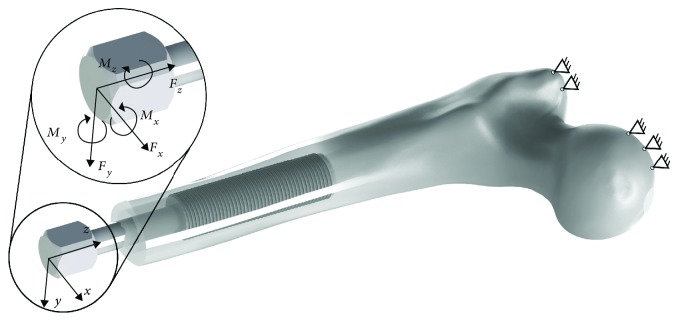
The implantation method, considered coordinate system for loads, load location, and the positioning of supports.

Analyses with the use of finite element method were performed using the Ansys Workbench 16.0 software (Ansys Inc.). Implants with appropriate threads were placed in the left femur model of an adult human (male, 44 years old, 85 kg mass, 185 cm height). In order to reflect postamputation conditions, the femur was cut in half of its length. Approximate bone shaft diameter was 32 ± 2 mm, while approximate marrow cavity diameter was 16 ± 2 mm and the length from the head to the amputation level was 237.5 mm. The implantation method reflects the positioning of the OPRA system [5, 8]. The length of the implanted part was 75.0 mm, while the initial immersion of the implant was 25.0 mm. The supports were set in the greater trochanter and femur's head areas. The exemplary implant-bone model is shown in Figure 2.

The analyses included two loading conditions. The first was the axial loading of the implant with a force of Fz = 1,000.0 N. A given load may occur during static load bearing exercises, that is, during rehabilitation process after implantation of an implant for bone-anchored prostheses. These exercises rely on loading a head of an implant with its user's own, most of the time, partial mass [29, 30]. The applied force occurs in case of extreme implant loading by a man of approx. 100 kg. The second loading method used in the analyses was taken from the experimental research of the OPRA system and corresponds to the highest forces that are generated on the implant's head during the gait for a patient of 61 kg [11, 31, 32]. These are obtained during the heel strike (*F*_*x*_ = 100.0 N; *F*_*y*_ = −20.0 N; *F*_*z*_ = 780.0 N; *M*_*x*_ = 30.8 Nm; *M*_*y*_ = −7.2 Nm; *M*_*z*_ = −2.0 Nm) and can lead to microcracks in bone tissues, which can result in the implant's loosening. The loads, in both analysed cases, were applied onto the head of the implant. In order to reflect the real conditions, a suitable coefficient of friction with a value of 0.4 was applied; as in primary stabilisation, the implant is not yet osseointegrated, which can lead to its micromovement within the bone tissues [32]. The reciprocal interaction of the implant and the bone was taken into account by applying the relevant contact elements.

To generate the implant-bone model, 10-node finite elements were used [33]. The authors focused on analysing the implant-bone interface. For this reason, the mesh was additionally densified in abovementioned contact to obtain the exact location of stress peaks generated in the bone during implant loading. The discretisation was performed until further densifying was not changing the results more than 3% obtained as Huber-Mises-Hencky stresses. The obtained models contained approx. 350,000 ± 25,000 finite elements with maximal edge length of 3.0 mm for the implant-bone model.

Orthotropic properties of the cortical tissue were considered in the research: *E*_*x*_ = 12 GPa (radial), *E*_*y*_ = 13.4 GPa (transverse), *E*_*z*_ = 20 GPa (longitudinal), *ν*_*x*_ = 0.376, *ν*_*y*_ = 0.222, *ν*_*z*_ = 0.235, and *ρ* = 1910 kg/m^3^ [3, 14]. Due to the fact that the implant is placed primarily in the cortical bone, for simplification, the isotropic properties of the cancellous tissue (*E* = 0.96 GPa, *ν* = 0.3, and *ρ* = 630 kg/m^3^) were used [34]. Titanium alloy (Ti6Al4V) was set as implants' material (*E* = 0.96 GPa, *ν* = 0.3, and *ρ* = 630 kg/m^3^) [5, 9].

## 3. Results

### 3.1. Comparative Analysis of Standardised Threads

In the first part of the research, a comparative analysis of the biomechanical functionality of the included standardised HA, HC, and HD threads was conducted.

**Figure 3:**
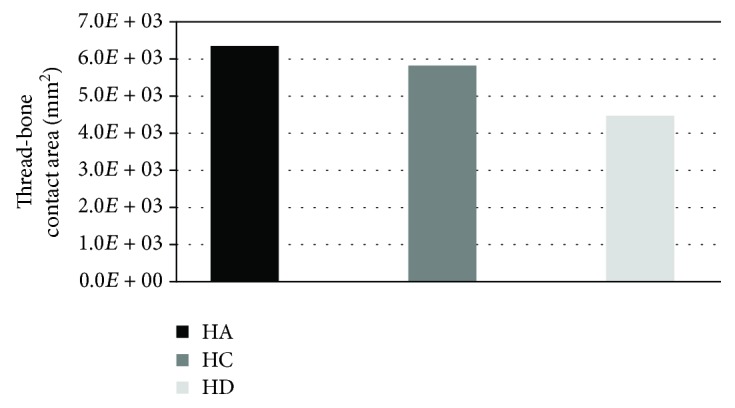
Differences in thread-bone contact area using standardised threads.

The first analysed factor was thread-bone contact area (Figure 3). According to Hansson and Werke and Lee et al., this parameter can then significantly affect the local concentrations of bone stresses arising during loading of the implant [17, 18]. Moreover, wider contact area should increase the effectiveness of implant-bone connection in secondary stability, by allowing the bone tissue to overgrow through a larger surface.

**Figure 4:**
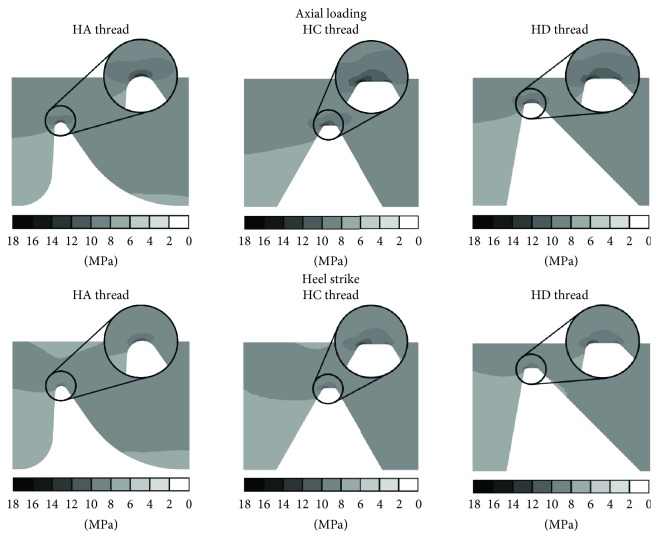
Equivalent stress patterns (MPa): cross section through stress peak location.

Another and at the same time the main analysed factor was stress patterns. According to Hansson and Werke, stresses concentrated in localised area are a direct cause of local bone tissue damage [17]. The obtained results were presented as cross sections through the axis of implant-bone connection and the location of stress peak formed during implant loading (Figure 4). Stress patterns created during the heel strike differed slightly from those resulting from axial loading of the implant, while the location of stress peaks remained the same.

**Figure 5:**
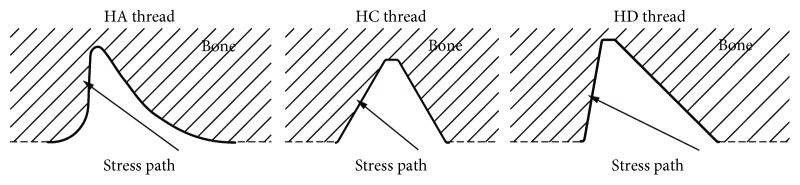
Stress paths to define stress profiles at thread-bone interface.

Stress profiles were set in order to increase the precision of conducted analyses. For this purpose, suitable paths (Figure 5) were determined, for which the equivalent and directional stresses were designated.

**Figure 6:**
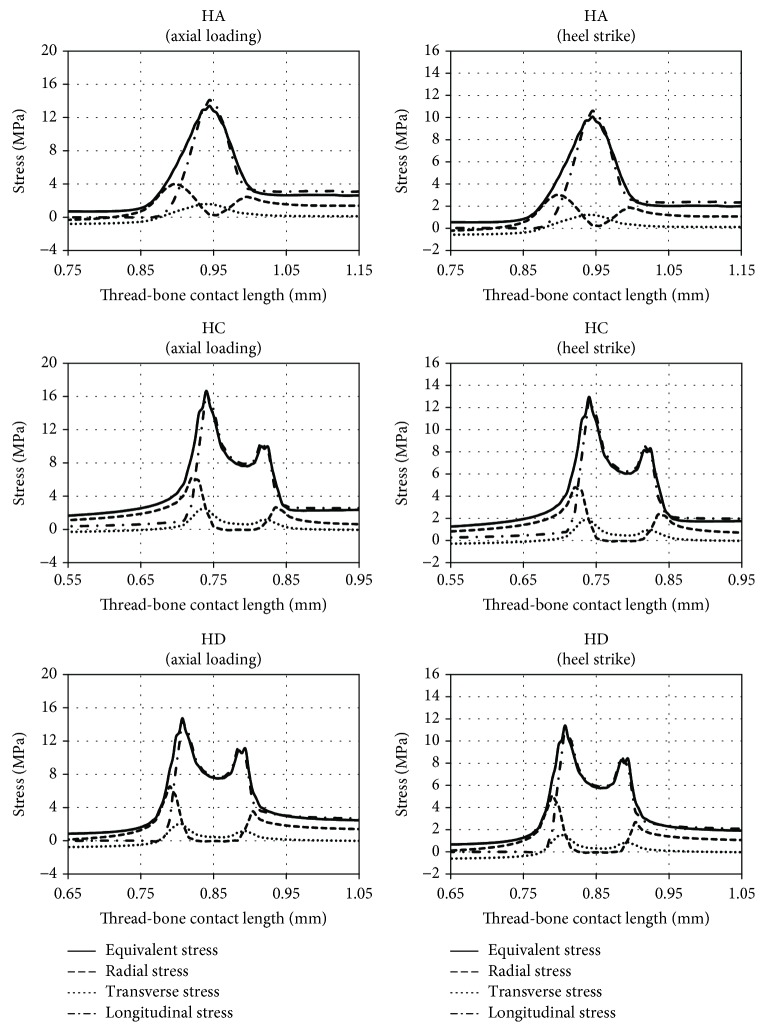
Individual stress profiles at the thread-bone interface during different loading types—the profile was limited to the section of stress peak formation (above and below of the presented values of the thread-bone contact length the stress profile changed linearly).

The obtained profiles allow for better understanding of the stress patterns presented in Figure 4. The profiles are presented in Figure 6.

**Figure 7:**
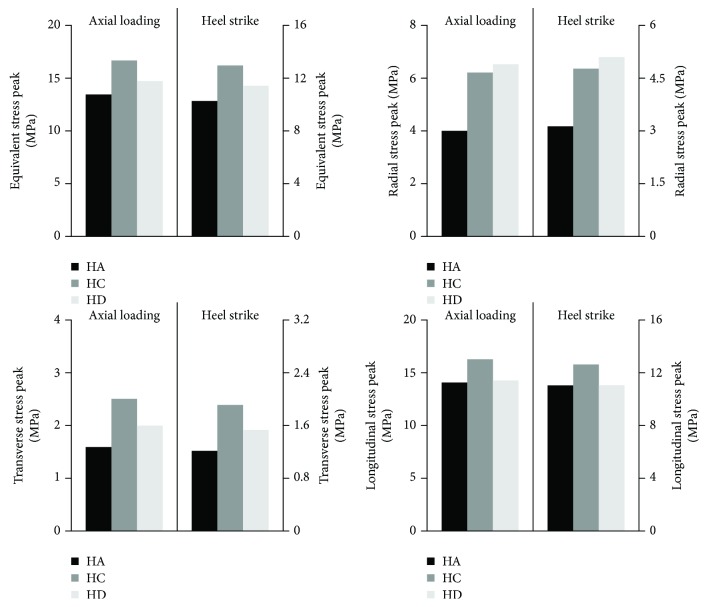
Stress peak values obtained during the two loading types.

The authors additionally analysed the stress peak values for both loading methods. The obtained results are included in Figure 7.

### 3.2. Comparative Analysis of Modified HA Thread

In the second part of the research, the influence of changes in parameters of the most suitable thread defined in the first part was examined (HA thread). In both parts, the same mesh parameters, loadings, and boundary conditions were used.

**Figure 8:**
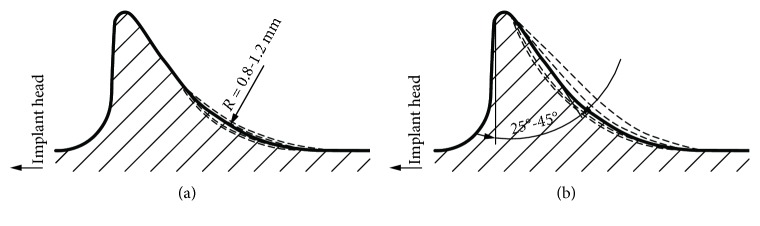
Thread geometry modifications used in the study. (a) Rounding arc. (b) Flank angle.

The impact of changes in the two thread's parameters was examined (Figure 8): the rounding arc (r1) and the flank angle (*α*). The stress distribution around the thread of the implant, presented in Figures 4 and 6, suggests that these two parameters have the main influence on the load transferring efficiency. Other thread's parameters should have then primarily impact only on implant's anchoring effectiveness in the cortical bone. Impacts of the 4 modifications of the rounding arc (0.8 mm, 0.9 mm, 1.1 mm, and 1.2 mm) and 4 modifications of the flank angle (25°, 30°, 40°, and 45°) on generated stresses were analysed. Obtained values were compared to values obtained in the first part of the research for HA thread type with standardised parameters (rounding arc = 1.0 mm and flank angle = 35°). Lastly, a comparative analysis of 25 thread geometries was performed.

**Figure 9:**
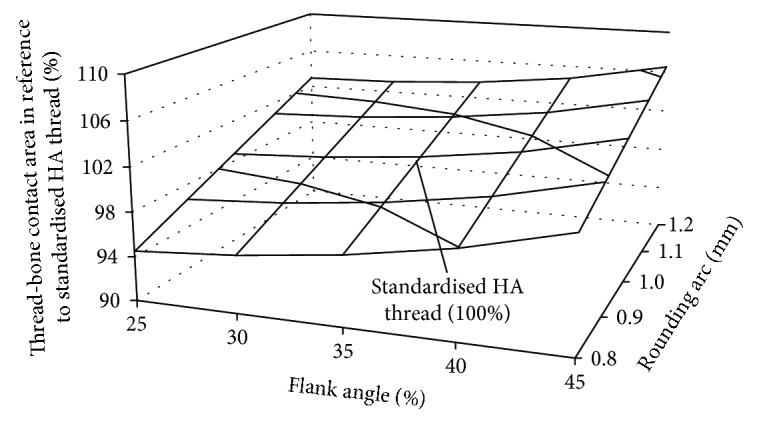
The influence of flank angle and rounding arc on the thread-bone contact area.

As in the first part of the results, the thread-bone contact area was determined in the form of the influence of changes in the flank angle and the rounding arc on analysed factor (Figure 9).

**Figure 10:**
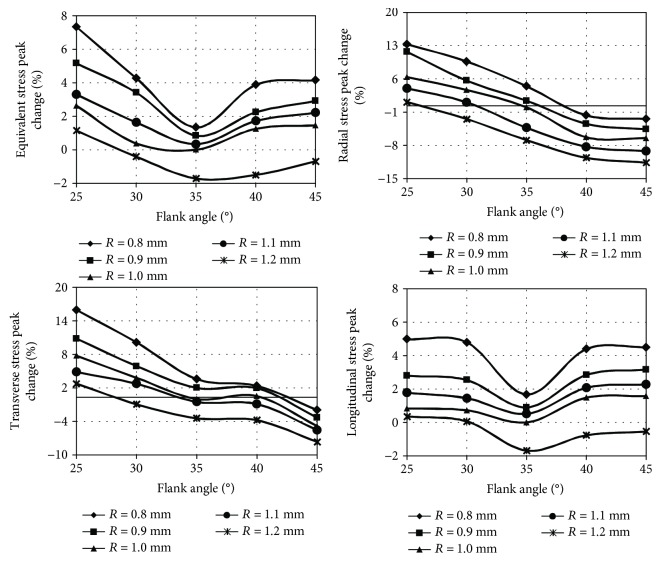
The influence of flank angle and rounding arc on stress peaks during implant axial loading with a force of 1000 N.

**Figure 11:**
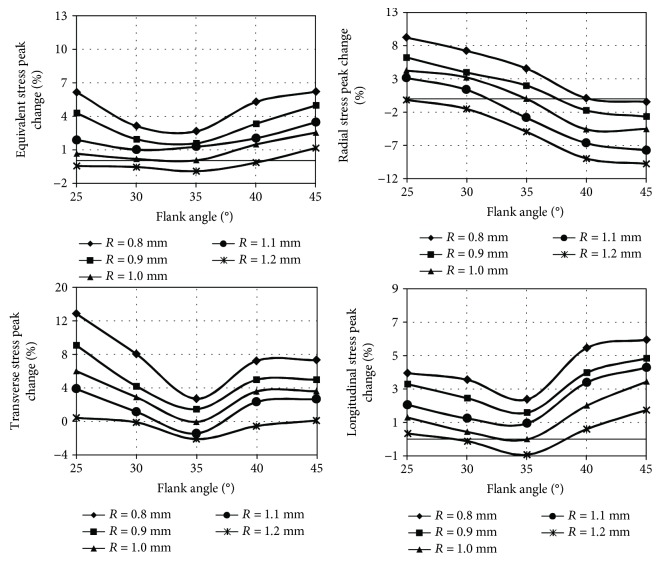
The influence of flank angle and rounding arc on stress peaks during implant loading with loads generated during heel strike.

The stress peak localisation did not change and remained similar as presented in Figure 6 despite the introduced constructional changes of implant-bone connection. The results of stress peak values obtained for axial loading are shown in Figure 10, while for heel strike in Figure 11.

## 4. Discussion

The following article presents a comparison of the efficiency (in terms of stress distribution in bone tissues, generated while loading of the implant) of standardised threads (shallow thread HA, symmetrical thread HC, and asymmetrical HD) in implants for direct skeletal attachment of limb prosthesis [27, 28]. Currently, there are no studies that clearly determine the optimal shape of the thread for use in this type of implants. While choosing a suitable type of the thread in order to obtain proper implant-bone connection, researchers can base on studies of dental implants that use this anchoring method [17, 25, 26]. However, these implants use various types of threads from symmetrical-, trapezoidal-, to circular-shaped, which, due to their specific shape, can be used only in the mandible and maxilla. The aim of the conducted study was to determine the optimal shape of the thread for the implants for direct skeletal attachment of limb prosthesis and modify proper parameters to obtain its maximum efficiency.

One of the closest studies to the one presented by the authors is the analyses conducted by Hansson and Werke [17]. However, the authors made an attempt to simulate implant-bone connection closer to in vivo conditions. Hansson and Werke assumed frictionless contact between the implant and the bone as well as simplified cortical bone to isotropic material. Unlike them, the authors considered the bone as an orthotropic material and used the appropriate coefficient of friction [3, 14, 32]. Due to the problem with convergence during calculation, Hansson and Werke applied inflated Young's modulus of the cortical bone. The authors used appropriate and actual Young's modulus value [35, 36]. Because of the adopted simplifications, Hansson and Werke focused on principal stress, while the authors of the paper decided to examine equivalent Huber-Mises-Hencky as well as directional stress peaks. Analysing the location of individual stress peaks may contribute to a better understanding of the mechanics of connection between threaded implants for direct skeletal attachment of limb prosthesis and bone tissues. Additionally, Hansson and Werke in their analyses considered a single implant member, while the authors of the presented paper analysed a complete model of the implant placed in the femur. This method greatly increased the computing time; however, it led to obtaining more appropriate load transfer from implant into bone tissues. The use of the described parameters allowed the authors for a better reflection of the actual implant-bone connection. As Hansson's simplification, the authors took into consideration the full contact of the implant with the bone.

### 4.1. A Comparison of the Effectiveness of HA, HC, and HD Threads

All stress peaks, equivalent and directional, were created in the area of the thread crest, which suggests that it is a region of possible bone resorption which occurs due to bone overloading. Obtained results are confirmed in other finite element and experimental thread analyses [17, 25, 26]. The described feature was observed in all types of analysed threads and can be the reason for the loosening of implants in the bone tissues. It leads to the necessity of its removal, which was reported in clinical experiments [17–21]. Moreover, the results obtained for axial and heel strike loadings present similarities both in the case of stress peak values as well as stress patterns and profiles. Therefore, it can be assumed that axial force is the main factor in the generation of stress peaks.

On the basis of the obtained results, it can be concluded that the optimal thread type (from the analysed types) for use in implants for DSA of limb prosthesis is the HA thread. It is determined due to the lowest equivalent and directional stress peak values that are generated in the bone during implant loading from all analysed thread-bone connections. Furthermore, high-value stresses generated as a result of stress peak decrease in the HA thread type much faster than in other types. Additionally, the use of HA threads significantly reduces radial (4.0 MPa and 6.5 MPa for HA and HD, respectively, during axial loading and 3.1 MPa and 5.1 MPa during heel strike) stress peaks. This is especially important due to the susceptibility of the bone on shear stress, which is one of the main reasons for bone resorption around the threaded implant. What is more, in the analysis of stress profiles generated in the case of HC and HD threads, there can be noted a second stress peak (equivalent and directional) with about 30% lower value. This indicates the existence of a second potential bone resorption site due to a sudden stress increase.

The results suggest that the least favourable is the HC thread around which the equivalent stress peak was created with the highest value reaching approx. 16.6 MPa during axial loading and 12.9 MPa during heel strike—HA generated 13.4 MPa and 10.3 MPa, while HD generated 14.7 MPa and 11.4 MPa, respectively, for axial and heel strike loadings. However, the HC thread provided lower radial stress peak (6.2 MPa for axial loading, 4.7 MPa for heel strike) than the HD thread (6.5 MPa for axial loading, 5.1 MPa for heel strike).

In the analyses, the impact of thread-bone contact area on stress peaks was not observed. Nevertheless, it should be remembered that higher contact area between an implant and a bone allows for obtaining a more stable secondary connection. For this reason, the implant construction should consider a thread that provides both, small values of stress peaks and a correspondingly large implant-bone contact area.

### 4.2. A Comparison of the Effectiveness of Modified HA Threads

The modification of the rounding arc by 0.2 mm and the flank angle by 15° from the standardised values (1.0 mm for the rounding arc and 30° for the flank angle) allows changing the thread-bone contact area by approx. 6.5%. This variation modifies the implant-bone interface affecting the probability of achieving adequate osseointegration during secondary stabilisation.

In the case of axial loading, the highest values of analysed stress peaks were generated at a flank angle of 25°. Moreover, the longitudinal stress peak was equally unfavourable at a 45° flank angle. Similar dependencies were observed in the case of heel strike, but in this case, equivalent and longitudinal stress peaks were the highest with a flank angle of 45°. Additionally, as in the axial loading, the radial and transverse stress peaks were characterised by the highest value at a flank angle of 25°. The lowest values of equivalent and longitudinal stress peaks during axial loading, as well as heel strike, were produced at a standard flank angle of 35°, while the lowest values of radial and transverse stress peaks were produced at 45°. Decreasing rounding arc caused an increase in the value of all stress peaks in each of the analysed cases.

The most favourable stress peak reduction was obtained by modifying only the rounding arc increasing it by 0.2 mm, leaving the flank angle at a normalised value of 35°. The described modification in the case of axial loading allows to reduce the equivalent stress peak by 1.7%, radial stress peak by 6.9%, transverse stress peak by 3.5%, and longitudinal stress peak by 1.7%. In the case of heel strike, this modification reduces the equivalent stress peak by 1.0%, radial stress peak by 4.9%, transverse stress peak by 2.1%, and longitudinal stress peak by 0.9%.

### 4.3. Limitations of the Study

The assumptions considered by the authors are characterised by certain simplifications in relation to real conditions. Among them there can be specified full contact of the implant with the bone, orthotropic and isotropic properties of the cortical and cancellous bones, respectively, or omitting the influence of body fluids as well as bacteria that often settle in the implant-bone interface. The impact of the stump muscles was also ignored.

## 5. Conclusions

The most appropriate standardised thread for use in implants for direct skeletal attachment of limb prosthesis is the HA thread type. It provides the lowest stress peaks' values and the most favourable stress pattern and profile.

The modification of the standardised HA thread by reducing the rounding arc by 0.2 mm allows for the reduction of the stress peak values in relation to the stress peaks generated in the bone around the standard HA thread. However, it should be remembered that this reduction also reduces the thread-bone contact area by 3.7%, which may decrease the probability of achieving proper osseointegration in secondary stabilisation.

The presented dependencies allow for the selection of a suitable thread for implants for the direct skeletal attachment of limb prosthesis and may lead to further research on the thread directly dedicated to implants for bone-anchored prostheses.

## Data Availability

No data were used to support this study.
